# Cytokine storms, evolution and COVID-19

**DOI:** 10.1093/emph/eoab005

**Published:** 2021-02-04

**Authors:** Joe Alcock, Alix Masters

**Affiliations:** 1 Department of Emergency Medicine, MSC11 6025 1, University of New Mexico, Albuquerque, NM 87131, USA; 2 Sidney Kimmel Medical College at Thomas Jefferson University, Philadelphia, PA 19107, USA

**Keywords:** SARS-CoV-2, Covid-19, cytokine storm, corticosteroids, immunity

## Abstract

Since the identification of severe illness caused by the novel coronavirus SARS-CoV-2, the role of the host immune system in causing disease has attracted widespread attention, along with intense interest in medical interventions that target the host immune response. A wide variety of agents have been proposed to treat a cytokine storm in coronavirus disease 2019 (COVID-19), but so far, only one class of medications, corticosteroids, has proved useful. In recent decades, experimental therapies for cytokine storms have been tried and mostly failed to help patients with severe sepsis and other infections. We summarize this history in order to frame expectations for novel interventions in COVID-19 and to bring an evolutionary medicine perspective to the concept of cytokine storms and their treatment.

## Lay Summary

Many treatments for COVID-19 are aimed at calming a cytokine storm, a dangerous immune overreaction to the infection. Treating cytokine storms has been tried for decades in sepsis and other viral illnesses, but these treatments most often do not work. We explain why cytokine storms should be rare, and what special evolutionary circumstances can cause them to occur.

## INTRODUCTION

With new and emerging infections, it can sometimes appear that the immune response does more harm than good. Excessive and dangerous immune responses have been cited in hantavirus pulmonary syndrome, Ebola virus [1], avian flu, H1N1 influenza [2], SARS1 and most recently, COVID-19 [3]. In the sickest COVID-19 patients, pathology has been described as an immune system gone awry, with an out-of-control inflammatory response driven by an apparent cytokine storm.

Cytokine storms—defined as a dysregulated, exaggerated and misdirected immune response accompanying excessive release of inflammatory cytokines—first appeared in the medical literature three decades ago in a report concerning graft versus host disease [4]. The term cytokine storms as applied to infectious disease has centered on viral illnesses [5] and influenza in particular [6, 7], along with septic shock caused by non-viral pathogens. Interest in cytokine storms has recently gained much attention with the COVID-19 pandemic [3].

A Science magazine profile included this quotation by the virologist Peter Piot, describing his personal experience with SARS-CoV-2 infection:

I turned out to have an organizing pneumonia-induced lung disease, caused by a so-called cytokine storm. It’s a result of your immune defense going into overdrive. Many people do not die from the tissue damage caused by the virus, but from the exaggerated response of their immune system, which doesn’t know what to do with the virus. I’m still under treatment for that, with high doses of corticosteroids that slow down the immune system [8].

Since that article was published, the corticosteroid dexamethasone has shown promising results in severe COVID-19 [9]. In the RECOVERY trial, infected patients requiring supplementary oxygen or mechanical ventilation who were randomized to dexamethasone had improved survival [9]. Since corticosteroids reduce inflammatory responses, the findings of RECOVERY appeared to validate the hypothesis that cytokine storms contribute to COVID-19 mortality.

The idea that excess inflammation kills COVID-19 patients is paradoxical because robust immunity has been linked with survival (i.e. in young patients and female patients), while impaired immunity has been associated with higher mortality (i.e. in immunocompromised patients and the aged) [10, 11]. Furthermore, immune overdrive should tend to be uncommon because of strong selective pressures to pare back deleterious immune responses over time. The observation that dexamethasone is less effective in less severely ill patients, along with the failure of other anti-cytokine agents in COVID-19, suggests that immune defenses in COVID-19 are complex and should be considered a double-edged sword. An immune response needs to be matched to the infectious challenge in order to maximize host fitness—too much or too little might result in the death of the host [2].

The history of immune modulating medications in infectious disease is instructive when considering treatments aimed at calming a cytokine storm in COVID-19. Some pharmaceutical interventions under study for COVID-19 have analogs that were previously used to treat sepsis and septic shock, with the guiding hypothesis that restraining hyperinflammation would benefit survival. However, despite the support of promising preclinical results and a clear-cut mechanistic rationale, the vast majority of immune modulating treatments in sepsis have not improved survival [12]. It has been crucially reported that the cytokine profile in plasma of severe COVID-19 infection does not differ significantly from acute respiratory distress syndrome (ARDS) and sepsis [13]. This is an important observation in that it tells us that previous research on ARDS and sepsis treatments is potentially relevant to COVID-19 cases.

Here, we critically examine the origin of the ‘cytokine storm’ concept and discuss how this notion influences patient treatment and research priorities. We describe the outcome of trials aimed at suppressing hyperinflammation in sepsis and other infections. Finally, we analyze this potential dysregulation of the immune system in the context of evolutionary medicine. When might we expect that the immune system, having evolved to protect us from infection, should instead go out of control and kill us?

## TREATING CYTOKINE STORMS

**Figure 1.**
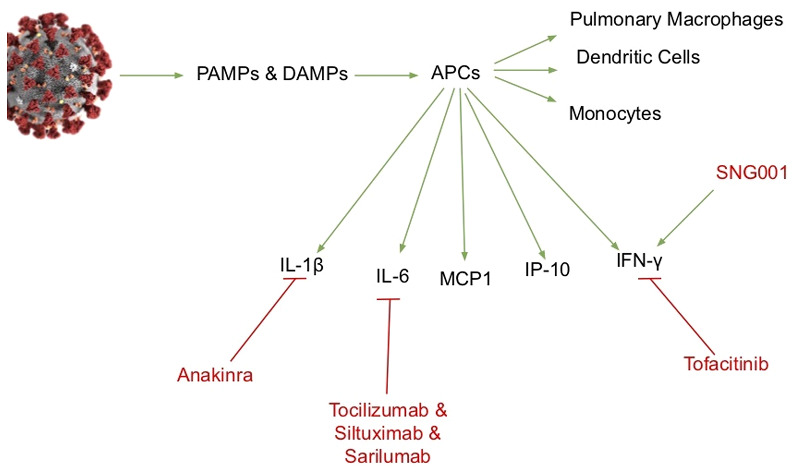
Cytokine targets of treatment for COVID-19. Antigen-presenting cells are a key source of pro-inflammatory cytokines and chemokines in COVID-19 infection. A variety of pharmaceutical agents that inhibit cytokines are under investigation.

Several investigators have proposed that hyperinflammation is a primary cause of severe COVID-19 and thus have advocated for therapeutic interventions against cytokine storms [3, 14]. A wide variety of immunosuppressive medications are being considered for COVID-19, including corticosteroids, Janus kinase inhibitors, and anti-cytokine treatments (Fig. 1). If excessive cytokine release is induced by COVID-19, as these investigators suggest, it follows that anti-cytokine treatments, such as inhibitors of the pro-inflammatory cytokines TNF-α and interleukin (IL)-6, should be beneficial [15].

### Anti-cytokine monoclonal antibodies

**Figure 2.**
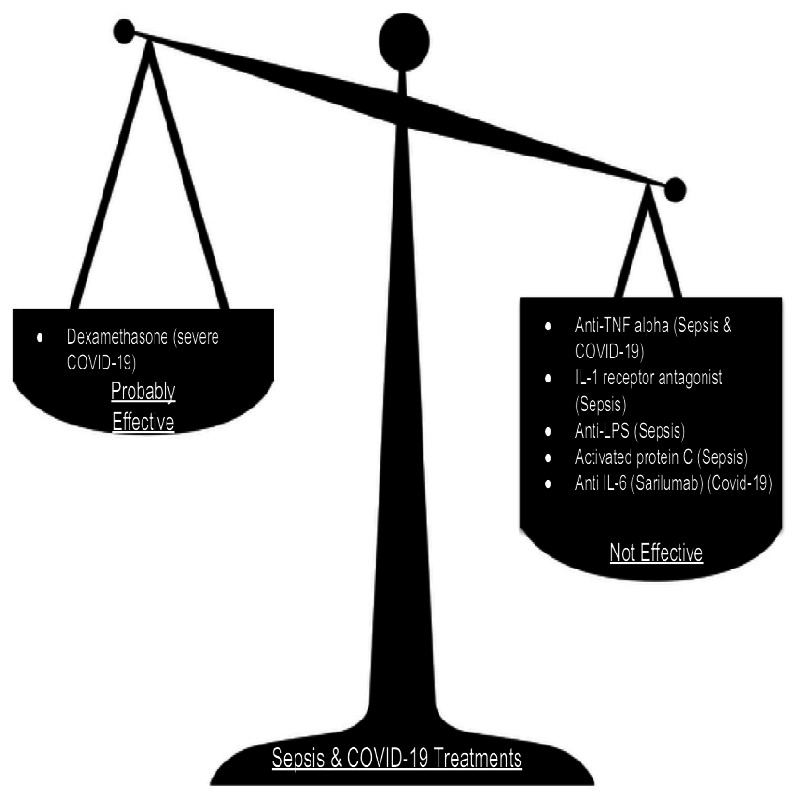
Anti-inflammatory treatments in sepsis and COVID-19. Evidence supports using the corticosteroid dexamethasone in severe COVID-19. However, in the vast majority of trials of human sepsis and COVID-19, anti-inflammatory medications have not improved survival.

Many immunomodulatory drugs being proposed for COVID-19 were originally developed for and tested in sepsis. However, early promising results of anti-cytokine treatments in sepsis led to disappointing large-scale randomized controlled trials in sepsis (Fig. 2). Many agents have subsequently found a role in treating chronic inflammatory conditions, including rheumatoid arthritis and Crohn’s disease.

Damas *et al*. [16] in 1992 pointed out that cytokine levels, particularly TNF-α, IL-1β and IL-6 were associated with mortality in severe sepsis. In particular, they wrote:

IL-6 correlated well with APACHE II score, and the mortality rate increased significantly in the group of patients who presented with IL-6 serum level above 1000 pg/ml.

These findings ushered in an era of intense interest in lowering IL-6, TNF-α and other pro-inflammatory cytokines in order to stave off sepsis mortality. Subsequently in 1996, Fisher et al. published a randomized controlled trial in *NEJM* of the tumor necrosis factor receptor fusion protein (TNFR: Fc), aimed at reducing TNF-α signaling [17]. In that trial, patients treated with TNFR: Fc unexpectedly showed increased mortality compared with placebo [17].

Other trials also had negative results, including the RAMSES trial of antibody treatments directed at TNF-α [18]. Reinhart *et al*. [18] wrote:

The repeated failures of even large sepsis trials to demonstrate more than a favorable trend in survival benefit with anti-TNF-α therapy raises the possibility that expectations for this therapeutic approach may have been exaggerated.

Because sepsis was not seen as an appropriate target for these drugs, agents developed out of this research program were repurposed for autoimmune diseases and are known collectively as disease-modifying antirheumatic drugs (DMARDs). This use comes with an important caveat. When compared with lacebo, DMARDs have been shown to increase the risk of severe infection [19].

A recent review surveyed the landscape of immune modulating drugs in COVID-19 and concluded that ‘there is no evidence of the beneficial impact of IL-6 inhibitors on the modulation of COVID-19’ [20]. One such drug, sarilumab has been shown to inhibit IL-6 mediated signaling and is approved for rheumatoid arthritis. On 1 September 2020, its manufacturers reported that the drug failed to reduce mortality or shorten hospital stays in COVID-19 [21]. Tocilizumab is another anti-IL-6 monoclonal antibody agent approved by the FDA for rheumatoid arthritis. The advanced phase III COVACTA study of tocilizumab in hospitalized patients with COVID-19 pneumonia failed to meet its primary endpoint of improved clinical status [22]. The COVACTA trial is one of five randomized trials on tocilizumab that were summarized in a recent *JAMA* editorial [23]. None of these trials reported a mortality benefit at 28 or 30 days. The majority did not meet their prespecified primary outcome measure for clinical efficacy [23].

### Corticosteroids

Before COVID-19, influenza was the best studied viral infection regarding the use of corticosteroids. Because levels of pro-inflammatory cytokines are elevated in severe cases of influenza, corticosteroids have been extensively used in critically ill patients with flu [24, 25]. Brun-Buisson *et al*. [26] showed no benefit to corticosteroids in H1N1 influenza A ARDS, and this observational trial suggested higher mortality from early corticosteroid use. A recent review of immunomodulatory agents for influenza concluded: ‘currently there are no immunomodulatory agents that have been conclusively proven to be of benefit in severe influenza’ [24]. A 2019 Cochrane meta-analysis suggested increased mortality in patients with influenza receiving adjunctive corticosteroids. This study—including 21 randomized controlled trials, including 15 involving the 2009 H1N1 influenza—found a significant association between corticosteroids and increased mortality (odds ratio) 3.90, 95% CI 2.31–6.60; *I*^2^ = 68%). That report also included a pooled analysis of seven studies suggesting an increased risk of hospital acquired infection which may be responsible for the increased incidence of death.

In contrast to these earlier trials, the recently published RECOVERY trial showed a reduction in mortality at 28 days among hospitalized COVID-19 patients who were randomized to oral or IV dexamethasone compared with placebo. Improved survival in the dexamethasone group occurred only in severe COVID-19 cases requiring supplemental oxygen or mechanical ventilation [9]. There was no survival benefit, and a possibility of harm, in patients with less severe infection [9].

Improved survival with corticosteroids in COVID-19 may occur in some adults with ARDS [27], raising the possibility that a corticosteroid benefit in COVID-19 may accrue mostly to ventilated patients with decompensated lung status. However, evidence for corticosteroids in ARDS is mixed [28, 29] and a recent secondary analysis of a 2015 randomized controlled trial involving 745 patients with ARDS showed no mortality benefit from corticosteroids [30]. In sepsis, corticosteroids have also shown mixed results, but the largest multicenter randomized controlled trial showed no improvement in short- or long-term survival [31].

In contrast to the RECOVERY trial, several observational studies involving COVID-19 patients with pre-existing corticosteroid use suggest potential harms from this class of medication. In COVID-19, systemic corticosteroids in patients with inflammatory bowel disease had a 6.9 increased odds of mortality [32]. Furthermore, a recent observational study found that patients with chronic obstructive pulmonary disease who were previously prescribed inhaled corticosteroid treatment were at an increased risk of death due to COVID-19 infection. Similarly, it found that asthma patients prescribed a high dose of inhaled corticosteroids were at increased risk of COVID-19 related death compared with those given a low or medium dose. Although these results could be explained by confounding factors such as comorbid disease severity, it does complicate the picture of corticosteroid use as generally protective [33].

Although the RECOVERY trial showed a benefit to corticosteroids in ventilated COVID-19 patients, a trend to increased mortality in lower acuity COVID-19 cases in the same trial raises questions about which patients are likely to benefit versus suffer harm from their use [9]. Whether steroids help or hurt, for which indications, in what doses, and at what time in the disease course, continue to be sources of controversy. However, it is important to heed the lessons of corticosteroid trials in other severe viral infections and sepsis. We should expect tradeoffs from drugs like corticosteroids that have powerful pleiotropic effects on the immune system.

## IMMUNE OVERSHOOT—EVOLUTIONARY CONSIDERATIONS

With the exception of corticosteroids in severe adult COVID-19, immune modulating drugs in sepsis and severe viral infections have been mostly ineffective or they have proven harmful. These observations suggest excessive immune responses may be more infrequent than commonly supposed. However, there remains evidence that occasionally immune systems do, in fact, overshoot. Several possibilities exist to explain immune dysregulation and self-harm.

### Smoke detector principle and immune brinksmanship

The smoke detector principle has been proposed as an explanation for responses that are out of proportion to the apparent threat. Biological systems can overshoot in various scenarios, such as panic, and in certain immune responses [34]. The smoke detector principle suggests that an optimally regulated system can produce events that are excessive, even sometimes maladaptive for an individual. The evolution of threat detection and response systems is expected to produce occasional over-reactions. A smoke detector that is useful for a house fire provides an analogy: to get this kind of reliability needed to protect us, we are willing to accept occasional false alarms. Using this analogy, Nesse and Schulkin [35] have argued that ‘inflammatory responses to infections are relatively inexpensive compared with the catastrophe that could result from an inadequate response’. Although we would expect that selection would disfavor needlessly costly or lethal immune responses (see Box 1), the smoke detector principle may explain some ostensibly excessive immune responses during severe infections [36]. Like a smoke detector, toll-like receptors are triggered by danger signals (alarmins) and pathogen-associated molecular patterns (PAMPs), generating an inflammatory cascade that can both help and harm the host. Activation of TLR-4 by bacterial lipopolysaccharide, e.g. is sufficient to cause life threatening sepsis syndrome even when live bacteria are absent [37]. Consistent with expectations of the smoke detector principle, blocking TLR4 activation by lipopolysaccharide fails to improve, and sometimes worsens, host mortality during infections [38, 39]. These observations suggest that recognition of lipopolysaccharide is an evolutionarily conserved response that confers an adaptive benefit, on average, to the host [40]. A key implication of the smoke detector principle is that blocking defenses, even those that appear excessive, can have negative unintended clinical consequences.

Immune brinksmanship is another proposal to explain the evolutionary persistence of apparently harmful immune responses [41]. In this model, the host undertakes a risky gamble when mounting an immune response against infection that involves substantial harm to both the host and the pathogen [41]. However, the host gambles that those harms will be disproportionately directed to pathogens. The analogy is one of trade sanctions in which a country might undertake an economically damaging stoppage of trade with a competitor, with the calculation that the competitor will bear the brunt of the injury. For immune responses during infections, selection acting on hosts is expected to minimize, but not eliminate, the costs suffered by hosts. A casino provides another analogy for immune brinksmanship. As in a casino, where the odds are in favor of the house, the odds of immune brinksmanship favor the host. However, in casinos the house sometimes loses. Immune brinkmanship would be most protective when there has been sufficient evolutionary time for selection to fine tune it. This is not true for novel SARS-CoV-2, and some deaths may represent an immune gamble gone bad.

### Mismatch

Mismatch occurs when organisms are subjected to novel environments that are different from the environments that their ancestors evolved in and that their physiology evolved to expect. One example is the modern use of antibiotics and the Jarisch Herxheimer (JH) reaction. The JH reaction was named after two dermatologists in 1902 who noticed worsening skin rashes in patients with syphilis treated with mercury compounds. When penicillin became the treatment of choice for syphilis, the JH reaction was typified by a rash, and also fever, hypotension and sometimes death. The immune-mediated response of JH is reported in other spirochete infections, including Lyme disease and relapsing fever, and is linked with sudden increase in pro-inflammatory cytokines, including tumor necrosis factor [42].

Immune overshoot caused by antibiotics is a consequence of the sudden unmasking of bacterial antigens caused by dying and dead spirochetes. Spirochete molecular patterns that are otherwise inaccessible to the host immune system [43] initiate widespread antibody and complement-driven immune responses, and an apparent cytokine storm. The JH reaction only occurs with exogenous antimicrobials, suggesting that this cytokine storm occurs because a mismatch involving modern medical treatment. This response also highlights the smoke detector principle, since we expect that evolution would tend to favor threat detection and effector systems that err on the side of being more sensitive.

### Mismatch and COVID-19—host switching

Inexperience of the human immune system with novel coronavirus is another mismatch that might lead to sub-optimal immune responses. Crespi [44] has argued that this mismatch is the primary explanation for the hyperinflammatory response to COVID-19. Bats are the proposed reservoir hosts for many emerging viruses; the original host of SARS-CoV-2 is thought to be a horseshoe bat. Humans may not have had sufficient time to evolve optimal strategies to cope with this bat-adapted virus [44]. When compared with humans, viral infections in bats often lack overt signs of disease. Bats may tolerate coronavirus infections better in part because they have higher constitutive expression of interferon (IFN)-α [45]. Two competing potential strategies exist for hosts to cope with infections—immune resistance and immune tolerance [46]. The idea of a fatal cytokine storm in COVID-19 dovetails with the notion that survival outcomes would be better if the host reduced anti-pathogen effort, a concept known as immune resistance, and instead engaged in a strategy termed immune tolerance [47, 48]. We might expect a host response that involves excess resistance and insufficient tolerance for a novel pathogen. This would be particularly the case if tolerance mechanisms are harder solutions that take more time to evolve than general-purpose resistance strategies. In addition, experimental and theoretical work suggests that that older organisms are at greater risk for mismatch-related pathology [49]. The notion that for virulent SARS-CoV-2 insufficient time has existed for selection to modulate immune responses with age-dependent effects deserves further study.

### Tradeoffs involving other pathogens

The host may face a tradeoff during COVID-19 infection when they are infected at the same time by multiple other pathogens. Coinfection also tends to select for higher virulence in parasites generally and might be expected to worsen the severity of COVID-19 [50]. Chronic infections and multiple infections in COVID-19 are commonly reported [51]. Many people are chronically infected with herpesviruses and other pathogens that are potent inhibitors of antiviral immunity [52], including the IFN responses that are a key defense against SARS-CoV-2. Consequently, some viral coinfections might worsen COVID-19 outcomes. Similarly, coinfection with bacterial or fungal pathogens may trigger maladaptive immune responses in COVID-19, in part because of tradeoffs between defenses against viral and bacterial infections. One such tradeoff is exemplified by *FUT2* gene loss of function mutations that are common in many human populations. These *FUT2* variants confer protection against influenza A and other viral pathogens at the cost of increased susceptibility to various pathogenic bacteria, including *Streptococcus pneumoniae*, reviewed in [53] (Box 1).

## CYTOKINE STORMS AND LIFE HISTORY THEORY

Most COVID-19 infections are minimally symptomatic and self-limiting. Some patient characteristics increase the risk of more severe manifestations. An evolutionary perspective may shed light on certain life history characteristics of patients who are most at risk for a dysregulated immune response, or a potential cytokine storm. In this section we build on the insights of McDade [56] and others who have proposed a population-based life-history perspective on immune development.

Children under 20 are less than half as likely to be susceptible to symptomatic COVID-19 infection than adults over 20 [57]. Children are also more often exposed to respiratory viruses than adults [58] and hence likely exhibit immune cross-protection from other coronaviruses [59]. Evidence has also shown that children have greater amounts of isolated lymphoid follicles and Peyer’s patches (containing naïve T cells and regulatory T cells) in their gut which diminish greatly over time [60]. This could help explain the greater sensitivity to ingested antigens (food allergies) seen in children compared with adults [61] and potentially increased susceptibility to specific inflammatory syndromes such as Kawasaki’s disease. For cytokine responses in particular, T-cell intracellular cytokines tend to increase with age in healthy children [62]. TNF-α concentrations in stimulated samples also increase with age. [63].

Some functional differences in the pediatric immune system can be explained by life history theory. Considering all sources of infection, pediatric infectious mortality is highest at age 0–1, and is relatively high in early childhood (age 1–5), compared with later ages. High infectious mortality in early life may drive selection for accelerated lymphocyte clonal evolution in infancy and early childhood [56]. Prioritization of early lymphocyte expansion is reflected in the size of the thymus gland, which is greatest in infancy and disappears completely by early adulthood [56]. This phenomenon, termed thymic involution, is in line with hypothesis that strong selective pressure very early in life, i.e. *in utero*, generates T cells that are in place before significant exposure to harmful microbes occurs [64].

Irrespective of the cause of death, natural selection has decreasing power with age, a relationship that is one explanation for the evolution of aging. The fitness benefit of an effective immune response of children is high until maturity, and then it declines with increasing age. (The same is true for any organ—functioning typically declines with increasing age after reproductive maturity). Inflammatory responses that are constrained by age-related organ decline might explain why certain inflammatory responses have higher amplitude in children and young adulthood. Fever exhibits this pattern [65]. IFN responses also decrease with age [66].

In addition to age, sex influences immunity and the risk of infection. Men have greater mortality from infection compared with women throughout the lifespan [67]. Increasing evidence suggests significant differences in immunity across sex. Studies dating to 1942 have shown that female mice are capable of producing more antibodies than male counterparts [68]. Furthermore, sexual dimorphism, in both adaptive and innate immunity, has been demonstrated with testosterone generally showing an immunosuppressive effect, whereas estrogen has shown an immunoenhancing effect [69]. Although behavioral differences may drive some sex-specific infectious mortality, male-biased infectious mortality begins in infancy when behavior is similar, suggesting a physiological basis for infection severity [67].

A variety of evolutionary hypotheses are proposed for these sexual distinctions in immunity, based on life history theory and sexual selection [70, 71]. Sex differences in reproductive strategy are proposed to underlie differences in immune responses and infection vulnerability [71]. Males die more often from infection, reflecting a tradeoff between immune investment and anabolic and maintenance costs of muscle, driven by the hormone testosterone [72]. Relatively increased immune vigilance in females is protective against infection. However, higher immune vigilance is also hypothesized to potentially overshoot, contributing to disproportionate prevalence of autoimmune diseases in women [71].

Given these known immunological differences, it is significant to note that the populations that fare best with COVID-19 infection tend to be women and children [73, 74]. Meanwhile, patient groups known to have poorer immune responses, such as adult men and elderly adults, have shown to suffer more severe COVID-19 disease. These patterns in COVID-19 outcomes are paradoxical if we accept that excessively exuberant immune responses are responsible for severe COVID-19 cases. This leads us to believe that the emphasis on hyperinflammation as a treatment target may miss the mark. Instead, an inadequate or delayed initial immune response may set into motion events that lead to severe COVID-19 infection, and those impairments are more likely to occur in males and in older patients (Box 2).

## SUPPORTING INSTEAD OF SUPPRESSING IMMUNE RESPONSES

One key example of how the immune defenses of COVID-19 can be understood as a double-edged sword is in the contradictory research on IFN as a potential target of treatment. One JAK-STAT inhibitor, tofacitinib, is currently under study for COVID-19. Tofacitinib inhibits IFN-α *in vitro* [79], providing the basis for its potential use for a cytokine storm. Targeting IFN, though, raises a red flag. Inhibition of IFN has been shown to be deleterious in other infections and may be similarly problematic in COVID-19 [80].

Recent work on SARS-COV-2 has revealed that inhibition of IFN is a primary virulence strategy of the virus [44]. Like the original SARS-COV, non-structural proteins encoded by the SARS-COV-2 genome have the functional effect of reducing IFN early in infection. In a recent study comparing the virological differences between SARS-COV and SARS-CoV-2, SARS-CoV-2 was specifically found to potently antagonize IFN-I [81]. Additionally, patients with genetic polymorphisms that result in impaired IFN responses have higher mortality from COVID-19 [82]. These findings suggest that it is potentially dangerous to use a treatment that disables a key antiviral defense, acting in the precise mechanism of action as the virus itself.

IFNs induce a wide array of gene expression, including genes coding for the antiviral protein vipirin. These antiviral effector functions are important in the defense against multiple viruses [83]. In line with the antiviral defense function of IFN, intervening to augment or stimulate the IFN response early on in infection may have therapeutic effects. A small exploratory study of 77 patients infected with SARS-CoV-2 showed treatment with IFN-α2b shortened the duration of viral shedding [84]. Another Phase 2 trial showed that the addition of injectable IFN-β-1b, in combination therapy, was effective in suppressing the shedding of SARS-CoV-2 [85]. Further, the British pharmaceutical company Synairgen published results from a trial of a novel inhaled INF-β-1a drug, SNG001. Patients randomized to SNG001 showed a greater odds of disease improvement (based on WHO Original Scale of Clinical Improvement) compared with those receiving placebo [85].

These observations suggest that suppressive approaches to limiting inflammation in COVID-19 could have unintended consequences in some vulnerable patients. Instead of inhibiting these responses, supporting them may sometimes be a better strategy. This notion is in line with the argument offered by Remy *et al*. [86]. They write:

if SARS-CoV-2 infection is similar to other chronic inflammatory and immune suppressive diseases, such as sepsis, we argue that immune stimulants, and not anti-inflammatory agents, should be considered as the first-line treatment option.

## CONCLUSION AND FUTURE DIRECTIONS

A maladaptive host response in the setting of a novel COVID-19 infection is possible because of evolutionary novelty and mismatch, since selection has had insufficient time to modify host immune characteristics. However, available evidence does not suggest that dysregulated immunity, or cytokine storms in particular, present a promising target of treatment for most infections.

Cytokine storms are a conceptual frame, or hypothesis, that comes with testable predictions. The most important prediction that follows this hypothesis is that anti-cytokine therapies should increase survival in diseases where cytokine storms are thought to occur. Overall, recent trials have had mostly negative results from agents that reduce pro-inflammatory cytokines (Figure 2). The inability of these drugs to improve COVID-19 mortality in randomized controlled trials casts some doubt on the hypothesis that cytokine storms are responsible for lethal COVID-19. Targeting cytokine storms should perhaps be de-emphasized in favor of approaches that support host protective immunity [86]. Alternatively, future treatments might focus on inhibiting the pathogen, not the host immune response [86]. These strategies include targeting viral evasion of immunity and using antiviral agents that reduce damage attributed to widespread infection of tissues [87]. This logic supports the use of drugs like remdesivir, an agent that targets and inhibits RNA viral synthesis and was recently approved by the FDA to treat COVID-19 [88].

The philosopher George Santayana wrote: ‘Those who cannot remember the past are condemned to repeat it’. Evolution and the recent history of medical trials have played out on different timescales—and yet when considering immunomodulatory interventions, we seem to have amnesia when it comes to both. Because of the complexity of the immune system and the legacy of selection acting on it, we should not expect that throwing a wrench into the system will often provide a meaningful fix. Even when cytokine storms are believed to cause mortality, the majority of trials aimed at hyperinflammation over several decades have not produced meaningful improvements in survival. Cytokine storms, when and if they occur, need more than a mechanistic explanation; they need a special case exemption, and an evolutionary rationale—for example, the mismatch hypothesis proposed by Crespi [44].

## SUMMARY

Progress in understanding and treating COVID-19 and cytokine storms requires placing the disease in the appropriate historical and evolutionary context: most anti-cytokine interventions have failed to improve outcomes because natural selection has shaped these responses to maximize benefits and minimize costs. Applying life history theory to COVID-19 may prove useful in understanding the demographic patterns of disease and potentially identifying groups who might benefit from immune-directed treatments.

Immune defenses are well-developed complex systems that reflect millions of years of selection imposed by parasites, and by the fitness costs of the immune response and embedded tradeoffs. We need to be careful in assigning pathology (as in fever), or excess (as in cytokine storms) or dysregulation (as in sepsis) to these responses.

**Conflict of interest:** The authors declare that they have no conflicts of interest related to this work.

Box 1. Why out of control immune responses should be rare in the infected host

**Excessive immune responses face strong selection to reduce their costly and self-injurious effects.**

Encounters with pathogens are problematic for hosts because infection decreases the Darwinian fitness of the host. Decreased host fitness takes two forms (i) direct injury as a result of viruses, for instance, commandeering the replicative machinery of host cells to make more virus—and (ii) indirect costs which include the metabolic and resource costs of mounting an immune response, reduced expenditure on other fitness enhancing functions, and friendly fire tissue damage from the response itself [54]. Hosts can evolve a variety of strategies to reduce the fitness costs of infection. These include behavioral immunity—including avoidance behavior (social distancing) triggered by overt signs of disease. Hosts can also evolve immune resistance strategies to clear or eliminate infections—these immune strategies are typically met with counteradaptations on the part of fast-evolving pathogens, in an arms-race scenario that has been described as the Red Queen effect [55]. If host immune defenses are excessive, causing excessive resources and/or friendly fire damage, hosts would be able to reduce expenditure in those self-defeating responses and be rewarded with better survival and increased fitness. If available, this last option is the low hanging fruit, providing an easily evolved way for the host to mitigate reduced fitness during infections. Unlike arm-races, it does not elicit a compensatory evolved response of the part of the pathogen. It also allows the host to avoid reproductive or other costs of behavioral immune activation. For this reason, medical interventions targeting excessive immune reactions are unlikely to improve outcomes, unless special circumstances exist.

Box 2. Kawasaki disease and multisystem inflammatory syndrome in children

The Kawasaki disease-like illness, known as Multisystem inflammatory Syndrome in Children (MIS-C), may be an example of an immune overreaction from COVID-19. Descriptions of MIS-C were first reported in April 2020 during the peak of COVID-19 activity in Europe, when a significant population of children with hyperinflammatory shock was reported in England. As of 29 July 2020, the Centers for Disease Control in the USA had reported 570 cases of MIS-C with 99.1% of these patients having received a positive SARS-CoV-2 serology or RT-PCR test [75].

Although MIS-C shares features with Kawasaki disease, a rare immune disorder that damages blood vessels and the heart, key differences exist. Many more children with MIS-C present in shock compared with Kawasaki patients (50% vs 5%) [76]. Additionally, MIS-C patients have higher ferritin, D-Dimers, and triglycerides than in Kawasaki disease, suggesting a separate pathogenesis [77].

MIS-C appears to occur *after* the virus has been largely cleared by the immune system, indicating it may be a post-infectious phenomenon [78]. Based on the relationship of MIS-C to SARS-CoV-2 infection it has been suggested that the pathogenesis of MIS-C involves a post-infectious immune overreaction. This hypothesis has been supported by the fact that MIS-C mirrors severe COVID-19 complications in adult patients, which coincide with a decline of viral load and increased markers of hyperinflammation in the respiratory tract [76].

It is possible that MIS-C represents an overreaction of the immune system to the novel SARS-CoV-2 virus for reasons that fit within our evolutionary medicine framework. For one, the pediatric immune system has been shown to be so efficient at corralling SARS-CoV-2, that it may be more apt to overreact, causing autoimmunity in MIS-C as a late sequela. It is also possible that due to recent host-switching of SARS-CoV-2 from bats to humans, the pediatric immune system has not had sufficient time to evolve an optimal adaptive immune response to this novel virus.
